# Behavior and season affect crayfish detection and density inference using environmental DNA

**DOI:** 10.1002/ece3.3316

**Published:** 2017-08-24

**Authors:** Nicholas Dunn, Victoria Priestley, Alba Herraiz, Richard Arnold, Vincent Savolainen

**Affiliations:** ^1^ Department of Life Sciences Imperial College London Ascot UK; ^2^ Thomson Ecology Guildford UK

**Keywords:** American signal crayfish, aquatic invertebrates, environmental DNA, invasives, quantitative PCR

## Abstract

Although the presence/absence of aquatic invertebrates using environmental DNA (eDNA) has been established for several species, inferring population densities has remained problematic. The invasive American signal crayfish, *Pacifastacus leniusculus* (Dana), is the leading cause of decline in the UK's only native crayfish species, *Austropotamobius pallipes* (Lereboullet). Methods to detect species at low abundances offer the opportunity for the early detection, and potential eradication, of *P. leniusculus* before population densities reach threatening levels in areas occupied by *A. pallipes*. Using a factorial experimental design with aquaria, we investigated the impacts of biomass, sex ratio, and fighting behavior on the amount of eDNA released by *P. leniusculus*, with the aim to infer density per aquarium depending on treatments. The amount of target eDNA in water samples from each aquarium was measured using the quantitative Polymerase Chain Reaction. We show that the presence of eggs significantly increases the concentration of crayfish eDNA per unit of mass, and that there is a significant relationship between eDNA concentration and biomass when females are egg‐bearing. However, the relationship between crayfish biomass and eDNA concentration is lost in aquaria without ovigerous females. Female‐specific tanks had significantly higher eDNA concentrations than male‐specific tanks, and the prevention of fighting did not impact the amount of eDNA in the water. These results indicate that detection and estimate of crayfish abundance using eDNA may be more effective while females are ovigerous. This information should guide further research for an accurate estimation of crayfish biomass in the field depending on the season. Our results indicate that detection and quantification of egg‐laying aquatic invertebrate species using eDNA could be most successful during periods when eggs are developing in the water. We recommend that practitioners consider the reproductive cycle of target species when attempting to study or detect aquatic species using eDNA in the field.

## 1 INTRODUCTION

Freshwater ecosystems contain almost 10% of all described species despite covering less than 1% of the earth's surface (Strayer & Dudgeon, 2010). However, native species within these ecosystems are under threat from the introduction of alien invasive species (Dudgeon et al., 2006; Strayer & Dudgeon, 2010). Invasive species are now regarded as one of the most severe threats to biodiversity across the globe (Clavero & García‐Berthou, 2005; Rahel & Olden, 2008), and the number of invasions into freshwater is expected to continue to increase over the next few decades (Strayer, 2010).

An invasive species must establish a self‐sustaining population before it is able to spread beyond its original introduction site (Blackburn et al., 2011). Therefore, the early detection of populations during this stage is key to effectively managing biological invasions (Simberloff et al., 2013; Waugh, 2009). However, this requires detection at low densities, and current methods used to detect invasive species in freshwater, such as catching or trapping individuals, are unable to do this reliably (Jerde, Mahon, Chadderton, & Lodge, 2011; Mackenzie, Nichols, Sutton, Kawanishi, & Bailey, 2005).

The detection of macro‐organisms using environmental DNA (eDNA) involves extracting and quantifying the DNA present freely in an environmental sample (e.g., soil, air, or water). This technique is rapidly developing and has already proved to be more reliable than traditional methods in detecting freshwater species (Dejean et al., 2012; Dougherty et al., 2016; Goldberg, Pilliod, Arkle, & Waits, 2011; Jerde et al., 2011; Schmidt, Kéry, Ursenbacher, Hyman, & Collins, 2013; Sigsgaard, Carl, Møller, & Thomsen, 2015). Most of the current aquatic eDNA research is focussed on fish and amphibians (Rees, Maddison, Middleditch, Patmore, & Gough, 2014; Roussel, Paillisson, Tréguier, & Petit, 2015; Thomsen et al., 2012), as these readily shed DNA into the environment via mucus and skin cells (Klymus, Richter, Chapman, & Paukert, 2015). Emphasis has been placed on the use of eDNA for the detection of invasive (Dejean et al., 2012; Goldberg, Sepulveda, Ray, Baumgardt, & Waits, 2013; Klymus et al., 2015; Tréguier et al., 2014) and rare species (Biggs et al., 2015; Jerde et al., 2011; Laramie, Pilliod, & Goldberg, 2015). Although eDNA has been successfully used to estimate fish biomass in both streams and aquaria (Doi et al., 2015, 2017; Lacoursière‐Roussel, Rosabal, & Bernatchez, 2016; Takahara, Minamoto, & Doi, 2013; Takahara, Minamoto, Yamanaka, Doi, & Kawabata, 2012), it not clear whether it is reliable for aquatic invertebrates.

To be able to successfully detect and quantify the abundance of a species using eDNA in the field, it is important to first understand how detection is related to abundance in a controlled environment. However, there has been little work on estimating the biomass and abundance of invertebrate species in the field or aquaria. Detection of invertebrate species via eDNA is considered challenging as exoskeletons are thought to prevent the release of cells and extracellular DNA (Dougherty et al., 2016; Tréguier et al., 2014). Despite this, as methods become more sensitive, eDNA studies have been able to successfully determine the presence or absence of gastropods (Goldberg et al., 2013) and arthropods (Dougherty et al., 2016; Forsström & Vasemägi, 2016; Ikeda, Doi, Tanaka, Kawai, & Negishi, 2016; Tréguier et al., 2014) from water samples. There is also no study that investigates the impact of behavior on the amount of DNA in an environmental sample, which is a crucial consideration when attempting to progress from controlled experiments to sampling in the field. Here, we assess whether eDNA can be used to successfully detect and quantify population densities of the American signal crayfish, *Pacifastacus leniusculus* (Dana), which is invasive in the UK.

A number of freshwater crayfish are highly invasive, with an almost unprecedented 90% invasion success record into European inland waters (Holdich, Reynolds, Souty‐Grosset, & Sibley, 2009). The only native crayfish species in the UK, *Austropotamobius pallipes* (*Lereboullet*), has experienced declines of over 95% in some regions of England and overall declines of up to 80% across Europe. This decline has resulted in the species being classified as Endangered on the IUCN Red List of Threatened Species (Füreder et al., 2010). Although numerous factors have contributed to the decline of the species, including pollution, habitat loss, and climate change (Diéguez‐Uribeondo, 2006; Holdich et al., 2009; Smith, Learner, Slater, & Foster, 1996), the greatest threat comes from invasive crayfish species via the spread of the oomycete *Aphanomyces astaci* (Schikora), otherwise known as the crayfish plague (Diéguez‐Uribeondo, 2006; Holdich et al., 2009), which is listed as one of the “World's 100 worst invasive alien species” (Lowe, Browne, Boudjelas, & De Poorter, 2000).

The American signal crayfish is endemic to western North America and was introduced to the UK in the 1970s (Holdich, James, Jackson, & Peay, 2014). It is now the most widespread invasive crayfish species in Great Britain (Holdich et al., 2014) and its distribution covers almost the entire range of the native *A. pallipes* (see Fig. S1). Natural densities and biomass of *P. leniusculus* can vary widely, depending on the water depth, habitat variability, and flow rate, with estimates ranging from 1.73 to 310 g/m^2^ (Flint & Goldman, 1977; Guan & Wiles, 1996; Shimizu & Goldman, 1983). The species is oviparous and, after spawning in October, females attach the eggs to their pleopods (shown in Fig. S2) for development (Mason, 1970a). The females remain egg‐bearing (hereafter ovigerous) for 6–9 months (Capurro, Galli, Mori, Salvidio, & Arillo, 2015; Lewis, 2002; Nakata, Tanaka, & Goshima, 2004) and it has been suggested that the activity of females decreases when they are ovigerous (Bubb, Lucas, & Thom, 2002). Studies suggest that there is a social dominance hierarchy in *P. leniusculus*, with female residents retaining shelter possession against male intruders (Peeke, Sippel, & Figler, 1995), but there does not appear to be clear differences in activity levels between males and females regardless of the season (Bubb et al., 2002).

Populations of *P. leniusculus* reduce the abundance of macrophytes and invertebrates in water systems to a greater extent than their native counterparts (Moorhouse, Poole, Evans, Bradley, & Macdonald, 2014), which in turn reduces the growthrates and abundance of amphibians and fish (Twardochleb, Olden, & Larson, 2013). Although they are not considered a burrowing species in their native habitat, *P. leniusculus* is known to burrow into riverbanks in the UK (Guan, 1994), making the banks unstable and susceptible to collapse (Barbaresi, Tricarico, & Gherardi, 2004; Guan, 1994), which significantly increases maintenance and repair costs (Williams et al., 2010). *Pacifastacus leniusculus* is a natural chronic carrier, and thus a vector of, the crayfish plague (Diéguez‐Uribeondo, 2006) and makes individuals of *A. pallipes* more susceptible to predation by competitively excluding them from refuges (Dunn, McClymont, Christmas, & Dunn, 2009). The ability to detect and eradicate this species before it establishes in lakes and rivers will be pivotal to the conservation of *A. pallipes* and eDNA could be a crucial tool in achieving this.

The objectives of this study were (i) to assess whether eDNA can be used to successfully detect and quantify the density of *P. leniusculus* in aquaria and (ii) to examine what impacts biomass, sex, and fighting have on eDNA concentrations. We hypothesized that (i) the amount of eDNA in a water sample would increase as crayfish biomass increases. We also hypothesized that (ii) tanks where crayfish could fight would contain higher eDNA concentrations than those where crayfish were prevented from fighting and that (iii) there would be no difference in the amount of eDNA in the water between sex‐specific and mixed sex tanks. Here, we used DNA capture by precipitation and qPCR to test the above hypotheses. We also considered the implications of our results for eDNA surveys and the management of invasive species.

## 2 MATERIALS AND METHODS

### 2.1 Crayfish husbandry

American signal crayfish were purchased live from a local, fully licenced crayfish supplier (Crayaway, London, UK) in January 2017, and species identification was confirmed using Holdich and Vigneux (2006). Upon arrival, crayfish were placed into aerated sex‐specific holding aquaria in a greenhouse at 20°C. Crayfish were fed throughout the experiment every week with 1 cm^3^ cube of carrot or potato per individual following recommendations by Longshaw and Stebbing (2016).

### 2.2 Primers and probe

Primer pair 5′‐GGAATAGTTGAAAGAGGAGTGGG‐3′ and 5′‐ATCAACAGAAGCCCCTGC‐3′ were used to amplify an 88 base pair fragment of COI. A specific dual‐labeled qPCR probe (5′‐6‐FAM‐CTGGATGAACTGTTTATCCTCCTCTAGCA‐BHQ1‐3′) was added to the qPCR mixes to increase specificity and allow detection (Fig. S3).

A general specificity test for each oligonucleotide was run in NCBI BLAST (Altschul, Gish, Miller, Myers, & Lipman, 1990) and we checked in silico that our primers do not amplify DNA from other interacting species using NCBI Primer‐BLAST (Ye et al., 2012). All oligonucleotides were synthesized by Sigma‐Aldrich^®^ (Sigma‐Aldrich, Gillingham, UK).

DNA was extracted from *P. leniusculus* tissue using a DNeasy Blood & Tissue Extraction Kit (Qiagen^®^, Hilden, Germany). DNA concentration was measured using a NanoDrop 2000c Spectrophotometer (Thermo Fisher Scientific^®^, Waltham, MA, USA). All DNA extractions and PCR preparations were performed in a PCR‐free dedicated laboratory.

The limit of detection (LOD, the minimum amount of target DNA that can be detected in a sample, Tréguier et al., 2014; Biggs et al., 2015) and limit of quantification (LOQ, the lowest level of amount of target DNA that yields a probability of false negatives under 5%, Tréguier et al., 2014; Biggs et al., 2015) for the primers and probe were calculated by performing a qPCR using extracted DNA in a serial dilution ranging from 10 ng/μl to 10^−8^ ng/μl with eight replicates per concentration. All qPCRs were run on a 96‐well plate in an ABI Prism^®^ 7000 (Applied Biosystems^®^, Warrington, UK) using 12.5 μl of TaqMan^®^ Environmental Master Mix 2.0 (Life Technologies^®^, Carlsbed, California, USA), 1 μl of each primer (10 μmol/L), 1 μl of the probe (2.5 μmol/L), 6.5 μl of DNase‐free water, and 3 μl of template DNA. Quantitative PCR conditions were as follows: initial steps of 50°C for 5 min and 95°C for 10 min, followed by 55 cycles of 95°C for 30 s and 62°C for 1 min. Each sample of DNA was run in triplicate, and three nontemplate controls were added to each plate.

To test for qPCR inhibition, a 119 bp synthetic gene was designed along with primers and probe (Fig. S4) using Geneious (v.10.0.7, Kearse et al., 2012). Each sequence was run through NCBI BLAST (Altschul et al., 1990) to confirm it was not a naturally occurring sequence of DNA, and Primer‐BLAST (Ye et al., 2012) was used to test the primers for nonspecific amplification.

### 2.3 Experimental design

**Figure 1:**
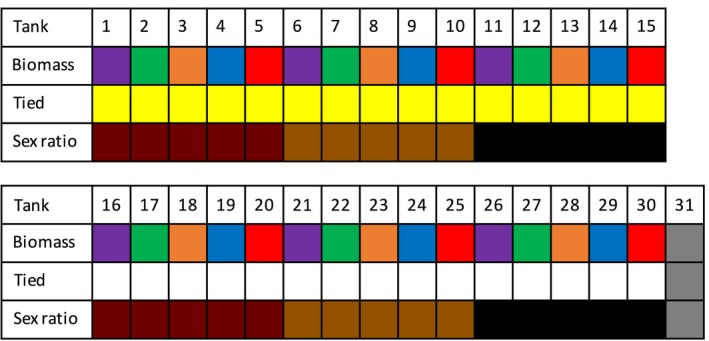
Experimental design: five biomass treatments (60 g, purple; 120 g, green; 200 g, orange; 300 g, blue and 600 g, red) were replicated six times, twice with 100% males (dark red), twice with a 50:50 sex ratio (brown), and twice with 100% females (black). Half of the replicates contained crayfish in which their chelae were tied (yellow), versus untied chelae (white). A control tank without crayfish (Tank 31) was also set up

We used a factorial design for our experiments. In total, 31 experimental 84 L tanks were set up in a greenhouse at 20°C, each containing 60 L water and two new overturned flower pots to act as refuges; each tank was aerated and covered with a lid. To investigate the impact of biomass on eDNA, each crayfish was weighed using a CL201 scale (Ohaus^®^, Parsippany, NJ, USA) and placed into the tanks at biomasses of 60 g, 120 g, 200 g, 300 g, and 600 g, respectively (±10 g, Fig. 1). The number of individuals in each tank and whether a given female was carrying eggs was recorded (egg biomass was not measured to avoid disturbing the females during the experiment). To investigate sex‐related differences in the amount of eDNA, the setup was reproduced twice with only males, twice with only females and twice with a 50:50 (±10 g) sex ratio in terms of biomass. Finally, to investigate the effect of fighting behavior on eDNA, crayfish chelae were tied with elastic bands in one of each of the sex ratio treatments (Fig. S2). Tanks were checked twice daily, and any dead individuals were removed and weighed. The final biomass of the tank used in the analysis was a daily average of the mass of the living individuals in the tank. A control tank without crayfish was set up alongside the experimental tanks.

### 2.4 Water sampling

After crayfish had been in the tanks for 11 days, 1 L of water was sampled from each tank by filling a 1 L Whirl‐Pak^®^ (Nasco, USA) bag using a 30 ml sterile ladle; a new pair of laboratory gloves were worn for the processing each sample. One tank was sampled at a time, and a new ladle was used for each tank. We checked that no eggs were present in the water samples. Each bag was mixed by inversion, and for each sample, six 50 ml centrifuge tubes (VWR, Lutterworth, UK), containing 35 ml of DNA preservative (33.5 ml absolute ethanol and 1.5 ml sodium acetate 3 mol/L), were filled to 50 ml with 15 ml of water using a sterile pipette. These were left to precipitate DNA at 4°C for 48 hr.

### 2.5 DNA extraction

The six subsamples from each tank were centrifuged at 14,000 × *g* for 35 min at 4°C, and the supernatant was discarded from each tube. Following Tréguier et al. (2014), the precipitated pellet in the first tube was resuspended using 360 μl of Buffer ATL, and this was mixed thoroughly by vortexing and then the solution was transferred to the second tube. This was vortexed and transferred to the next tube until the sixth tube contained a solution of all the DNA from the six subsamples. This was then transferred into a 2 ml tube and DNA was extracted from the solution using the DNeasy Blood & Tissue Extraction Kit (Qiagen^®^) following the manufacturer's spin column protocol; 100 μl of nuclease‐free water was used for the final elution of DNA. Once the water samples had been taken, crayfish were removed from the tanks and were killed by freezing at −80°C overnight. Aquaria were cleaned with a 10% bleach solution and rinsed thoroughly before they were set up again. The entire experiment was repeated twice.

### 2.6 Quantitative PCR

The samples were tested for inhibition by performing a qPCR with a solution containing 3 μl of the synthetic gene (10^−4^ ng/μl), 3 μl of extracted DNA from each tank respectively, 12.5 μl of Taqman Environmental Master Mix 2.0 (Life Technologies^®^), 1 μl of each primer for the synthetic gene, and 1 μl of probe. Thermal conditions were 50°C for 5 min and 95°C for 10 min, followed by 55°C cycles of 95°C for 30 s and 60°C for 1 min. All samples were run in duplicate, if one of these showed a quantification cycle value different to the expected value, the sample was considered inhibited and so a twofold dilution of the sample DNA was required (modified from Biggs et al., 2015).

Once the samples had been tested for inhibition, the extracted DNA was diluted if needed and amplified by qPCR using the *P. leniusculus* primers and probe. The qPCR solution contained 3 μl of template DNA, 12.5 μl of Taqman Environmental Master Mix 2.0 (Life Technologies^®^), 1 μl of each primer (10 μmol/L), and 1 μl of probe (2.5 μmol/L). Thermal conditions were 50°C for 5 min and 95°C for 10 min, followed by 55°C cycles of 95°C for 30 s and 62°C for 1 min. Samples were run in triplicate, and standards of known DNA concentrations, 10^−1^ to 10^−3^ ng/μl, were also run in triplicate on each plate along with three nontemplate controls containing nuclease‐free water instead of DNA.

### 2.7 Analysis

Mean target eDNA concentration was calculated from the triplicate samples, and analysis was only performed on quantities above the LOQ; an alpha‐value of 0.05 was set for all the statistical analyses in this study. Copy number was calculated using the online Thermo‐Scientific copy number calculator. Shapiro‐Wilk tests were performed to assess the normality of the data and, if required, the data were log_10_ transformed before being analyzed. All analyses were performed in RStudio v.0.99.903 (R Development Core Team 2016), and boxplots were created with the package “*ggplot2*”(Wickham, 2009).

From a maximal model, which included biomass (in g/L), whether individuals were taped or not, the sex ratio of the tanks, the presence of ovigerous females, and the proportion of ovigerous females, the minimum adequate model to explain the differences in log_10_ eDNA concentration was identified from analysis of covariance (ANCOVA) by stepwise substitution of linear models. The best fitting model was selected by running analysis of variance (ANOVA) tests between models. Linear models to determine the relationship between the biomass (in g/L) of *P. leniusculus* and the concentration of log_10_ eDNA in a sample were then made from subsets of the data depending on whether the aquaria contained individuals with eggs or not. The impact of eggs on log_10_ eDNA concentration in tanks holding crayfish at densities similar to natural densities (60 g, 120 g, and 200 g) was investigated using ANOVA. The impact of tying chelae was also investigated using ANOVA, and the differences between sex ratios were tested using an ANOVA and a post hoc Tukey honest significant difference pairwise comparison test.

## 3 RESULTS

A total of 544 crayfish were used, weighing an average of 28.2 g (5.5–52.6 g). A total of 22 (7.9%) individuals died during the first experimental run and six (2.3%) during the second, 122 (44.9%) of the total females were ovigerous.

### 3.1 Primer specificity and sensitivity

**Figure 2:**
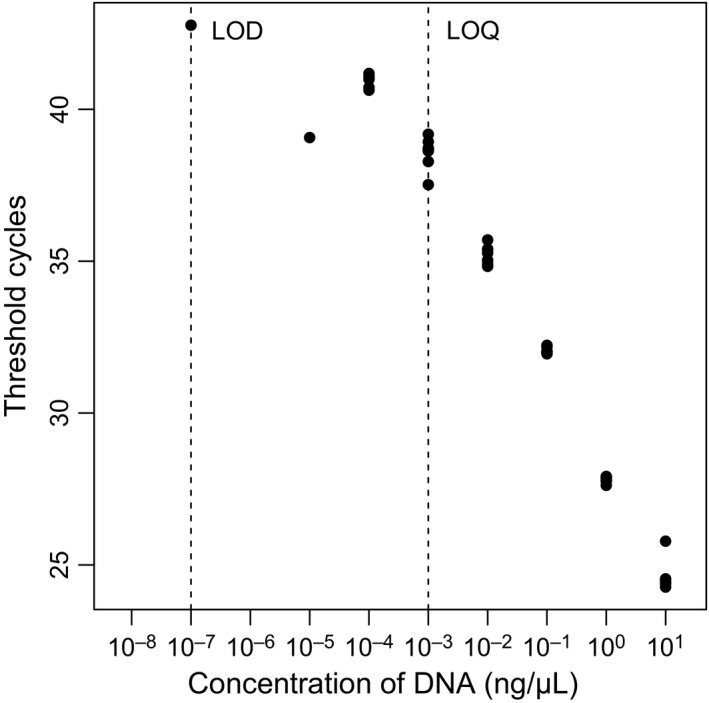
Limit of detection and limit of quantification for primers and probe (calculated from eight replicates of known concentrations of *Pacifastacus leniusculus* DNA; threshold cycles represent the PCR cycle in which the fluorescent signal was deemed to have exceeded the background level of fluorescence)

Although results from in silico testing indicated that some amplification of nontarget crayfish species would occur with the primers, the use of the specific probe makes it unlikely that nontarget DNA is amplified during qPCR. The LOD for this study was determined to be 10^−7^ ng/μl (one out of eight successful reactions), and the LOQ was found to be 10^−3^ ng/μl (eight out of eight successful reactions; Fig. 2).

Only one sample was inhibited, and *P. leniusculus* eDNA was detected above the LOQ in all but two samples (Table S1).

### 3.2 Impact of treatment on eDNA concentrations

**Figure 3:**
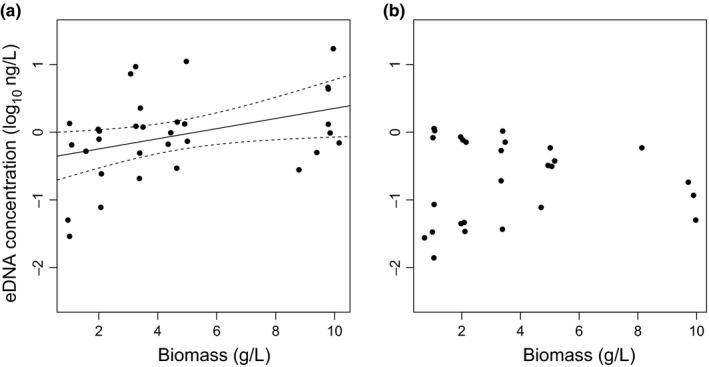
Eggs and eDNA. (a) Biomass of *Pacifastacus leniusculus* can be used to predict log_10_ eDNA concentration when there are ovigerous females in the aquaria using the regression *y *= 0.0750*x* − 0.397 (*r* ^2^ = .142), plotted as a black line with 95% confidence intervals as dashed lines (*n* = 32). (b) Log_10_ eDNA concentration cannot be predicted from biomass in tanks without ovigerous females (*n* = 27)

The minimum adequate model showed that target eDNA concentration increased with biomass and the presence of eggs (*F*_2,56_ = 10.1, *r* ^2^ = .265, *p* < .001), and the presence of eggs significantly increased the mean concentration of log_10_ eDNA by 0.608 ± se0.16 ng/L (*t* = 3.77, *p* < .001). The eDNA concentration significantly increased with the biomass of crayfish when the females in the aquaria were ovigerous (*F*_1,31_ = 4.97, *r* ^2^ = .142, *p* = .0334; Fig. 3a), whereas there is no significant relationship between eDNA concentration and biomass in tanks without ovigerous females (*F*_1,25_ = 0.0154, *p* = .902; Fig. 3b).

**Figure 4:**
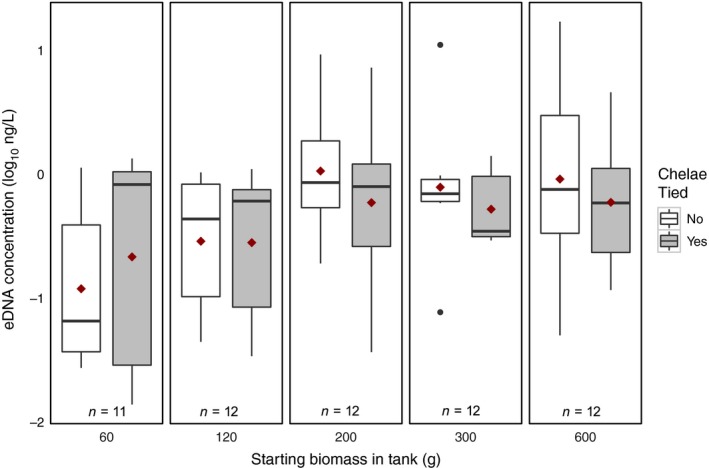
Behavior and eDNA. There was no significant difference in eDNA concentration between tanks in which crayfish had untied chelae (white boxes) and tanks in which crayfish had tied chelae (gray boxes), red diamonds indicate mean eDNA concentration

When the tanks containing unnaturally high densities of crayfish were removed from the dataset, the presence of eggs increased the log_10_ eDNA concentration by 0.586 ± 0.25 ng/L (*t* = 2.37, *p* = .0268) which, when back‐transformed, equates to an increase of 3.85 ± 1.8 ng/L. Tanks in which crayfish did not have their chelae tied had higher mean eDNA concentration in four of the five biomass treatments (Fig. 4); however, there was no significant difference between the means (*F*_1,58_ = 0.128, *p* = .722). There was also no significant difference between tied and nontied crayfish when tanks containing ovigerous females were removed from the dataset (Fig. S5). However, we witnessed one fight in one of the tanks, and it appeared to be the one with the highest eDNA concentration (17.1 ng/L). Sex ratio did have a significant impact on the amount of eDNA in the aquaria (*F*_2,56_ = 7.28, *p* = .0303; Fig. S6). Aquaria with only male crayfish contained, on average, 0.770 ± 0.49 less log_10_ eDNA than female‐only tanks (*p* < .05), but did not have significantly different amounts of eDNA to the mixed aquaria. Tanks with only females did not have significantly different concentrations of eDNA compared to mixed aquaria (Table 1).

**Table 1:** Results of a post hoc Tukey HSD pairwise comparison test showing the mean differences in log_10_ eDNA concentration (ng/L) between sex ratio treatments

	*n*	Difference	Adjusted *p*‐value
Male–Female	39	−0.770	0.00102
Mixed‐female	39	−0.450	0.0770
Mixed‐male	40	0.320	0.253

## 4 DISCUSSION

This study shows that eDNA can be used to detect and quantify *Pacifastacus leniusculus* in aquaria experiments and reveals that the presence of eggs significantly increases the amount of eDNA in a water sample. There was a high amount of variation in eDNA concentration between the tanks, which is consistent with previous eDNA quantification studies (Klymus et al., 2015; Pilliod, Goldberg, Arkle, & Waits, 2014; Turner et al., 2014). Males appeared to release significantly less eDNA than females, however this is likely to be a result of the ovigerous females. Our model suggests that there is a relationship between eDNA concentration and biomass when females are ovigerous, but there is no relationship between biomass and eDNA concentration in the tanks without ovigerous females. This suggests that mature crayfish do not release DNA at constant rate.

Our results indicate that the detection of crayfish at low abundances will be most successful when females are ovigerous. Crayfish eggs are comprised of soft tissue and are blanketed by a mucus secretion termed glair (Mason, 1970a). The eDNA is, therefore, likely to come from both the glair and the outer cell layer of the eggs themselves. While females are ovigerous, they clean eggs with their periopods and aerate them by fanning their pleopods (Mason, 1970b), and this is likely to further increase the amount of eDNA in the water and it was also observed that, in high density treatments, some eggs became loose.

In a study investigating biomass of fish in streams using eDNA, Doi et al. (2017) recorded the highest eDNA concentration at a site where there was nearby spawning activity and their models used to estimate abundance had significantly different slopes based on the sampling season (Doi et al., 2017). Dougherty et al. (2016) previously investigated the ability of eDNA to detect the invasive crayfish species *Orconectes rusticus* (Girard) in the USA and successfully detected the species in each lake it was trapped in as well as two lakes where no individuals had been trapped (Dougherty et al., 2016). However, the amount of eDNA at each site did not correspond to the estimated abundance (Dougherty et al., 2016). Similarly, Agersnap et al. (2017), Cai et al. (2017) and Larson et al. (2017) used eDNA to detect crayfish species in various environments, all recognizing that the link with abundance needs further validation.

One factor that was not taken into account here is the burrowing behavior of *P. leniusculus,* which could decrease the amount of eDNA available to sample in the water body. Tréguier et al. (2014) investigated the applicability of eDNA to detect the burrowing invasive crayfish species *Procambarus clarkii* (Girard) in France and only detected eDNA in 59% of the ponds where the species was known to be present (Tréguier et al., 2014). The concentrations of eDNA recovered were also all below the LOQ for the study meaning that reliable quantification was not possible (Tréguier et al., 2014). Ikeda et al. (2016) used eDNA to detect the burrowing crayfish species *Cambaroides japonicus* (De Haan) in streams in Japan. Crayfish eDNA was detected in all sites where individuals were captured by hand, and eDNA was also detected in two sites where no crayfish were caught (Ikeda et al., 2016). Quantification of abundance was not attempted in their study, and so further work on burrowing species is needed.

The variation in the amount of crayfish eDNA between tanks could be due to individual differences in excretion rates as a result of dominance hierarchies over food. It has been shown that the feeding behavior of bighead carp species in aquaria can increase the amount of DNA shed into the water by 10‐fold (Klymus et al., 2015). Here, the amount of food added to the aquaria was determined by the number of crayfish, although the amount of food any given individual consumed may have depended on the overall social hierarchy within the tank. Another reason for the variation may be due to contrasting injuries received by individuals as a result of fighting. However, our results indicate that the tying of chelae to prevent fighting in crayfish did not impact the concentration of eDNA in the aquaria. This may be a result of few encounters escalating to fights in the nontied tanks, resulting in a low frequency of injuries that would result in the release of DNA. It is noteworthy that the sample with the highest eDNA concentration (17.1 ng/L, Table S1) was one where a fight was witnessed, during which one crayfish sustained a serious injury, but as it survived, was kept in the tank. Given our experimental design, however, lack of fighting behavior prevented conclusions on whether or not fighting behavior and prevention of fighting impacted on the amount of eDNA in the water.

Our study used only relatively small crayfish, with an average mass of 39.2 g, whereas individuals of *P. leniusculus* can grow up to 110 g (Lewis, 2002). Aquaria were also kept 20°C, which is higher than natural UK winter temperatures (Bubb et al., 2002). As temperature has been shown to affect both the activity of crayfish (Bubb et al., 2002) and the amount of eDNA released into water by fish (Takahara et al., 2012), this may have impacted the amount of eDNA released into the tanks.

Our results have potential implications for further management research. Once invasive crayfish have established in an area, populations are very hard to control (Gherardi, Aquiloni, Diéguez‐Uribeondo, & Tricarico, 2011; Hein, Vander Zanden, & Magnuson, 2007). However, as biological control methods are improving (Freeman, Turnbull, Yeomans, & Bean, 2010; Peay, Dunn, Kunin, Mckimm, & Harrod, 2014; Sandodden & Johnsen, 2010), early detection of invasive species will be key to achieving eradication.

We show that *P. leniusculus* can be successfully detected in aquaria using eDNA. The LOQ for the set of primers and probe used in this study is higher than that reported in other eDNA studies on crayfish (Table 2), this may be due to the longer amplicon used here. However, there is clear need to standardize LOQ and LOD definitions and calculation methods for eDNA studies (Agersnap et al., 2017). We show that eDNA related to the eggs, rather than the animal itself, may increase the probability of successfully detecting the presence of crayfish, this is especially useful given the need for early detection in combatting invasive species. It might also be the case that without egg‐bearing females, it will not be possible to adequately infer population density from the eDNA concentration. Also, females of other decapods, both marine and freshwater species, bear eggs on their pleopods (Hazlett, 1983), and egg‐carrying is a common phenomenon in freshwater invertebrates (Pennak, 1985). The vast majority of amphibian species are also oviparous (Blackburn, 1999). We conclude that detection rate from eDNA could be higher for any oviparous aquatic species if water samples are taken during seasons eggs have been laid into the external environment. Further research is needed in this direction, particularly when focussing on oviparous species at low abundances. Nevertheless, we recommend that the reproductive cycle of the target species should be considered carefully to maximize detection and accuracy of the inferred density.

**Table 2:** Studies on crayfish eDNA using the COI gene, with limit of quantification (LOQ) and limit of detection (LOD) when reported. Note that Agersnap et al. used different definitions and methods for the determination of LOQ and LOD compared to the other studies

Crayfish species	References	COI amplicon size (base pairs)	LOQ (ng/uL)	LOD (ng/uL)	Main conclusion
*Procambarus clarkii*	Tréguier et al. (2014)	65	10^−4^	10^−8^	Detection successful; DNA amounts below LOQ
*Orconectes rusticus*	Dougherty et al. (2016)	128	Not reported	Not reported	Detection successful; poor correspondence between eDNA copy number and relative abundance
*Cambaroides japonicus*	Ikeda et al. (2016)	124	Not reported	Not reported	Detection successful; DNA quantification not attempted
*P. clarkii*	Cai et al. (2017)	65	10^−4^	10^−8^	Detection successful; positive correlation between eDNA concentration and crayfish count
*O. rusticus and Pacifastacus leniusculus*	Larson et al. (2017)	128 (*O.r*.) 184 (*P.l*.)	Not reported	Not reported	Detection successful; weak relationship between eDNA copy number and relative abundance
*Astacus astacus, P. leniusculus, Astacus leptodactylus*	Agersnap et al. (2017)	65	~1.7 × 10^−4^ ~1.7 × 10^−4^ ~1.7 × 10^−4^	~7 × 10^−5^ ~7 × 10^−5^ ~7 × 10^−5^	Detection successful; assays need further validation
*P. leniusculus*	This study	88	10^−3^	10^−7^	Detection successful; significant relationship between eDNA concentration and biomass of ovigerous females

## CONFLICT OF INTEREST

None declared.

## AUTHORS' CONTRIBUTIONS

ND, VP, and VS designed the research under VS supervision; ND ran the experiments and collected the data; AH helped with methods; ND wrote the initial manuscript; all authors contributed to the final text.
