# Supplementary figures and images for: Behavior and season affect crayfish detection and density inference using environmental DNA
Source: Ecol Evol. 2017 Aug 24;7(19):7777–85. doi: 10.1002/ece3.3316 (PMC5632632; doi:10.1002/ece3.3316)

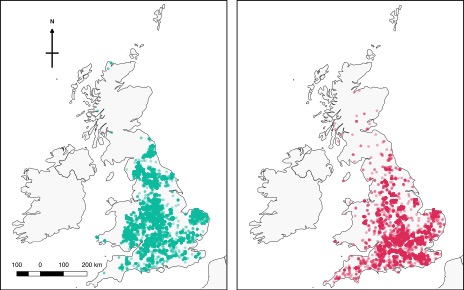

Supplement: Supplementary file 1 [file ECE3-7-7777-s001.jpg]

**(a)**

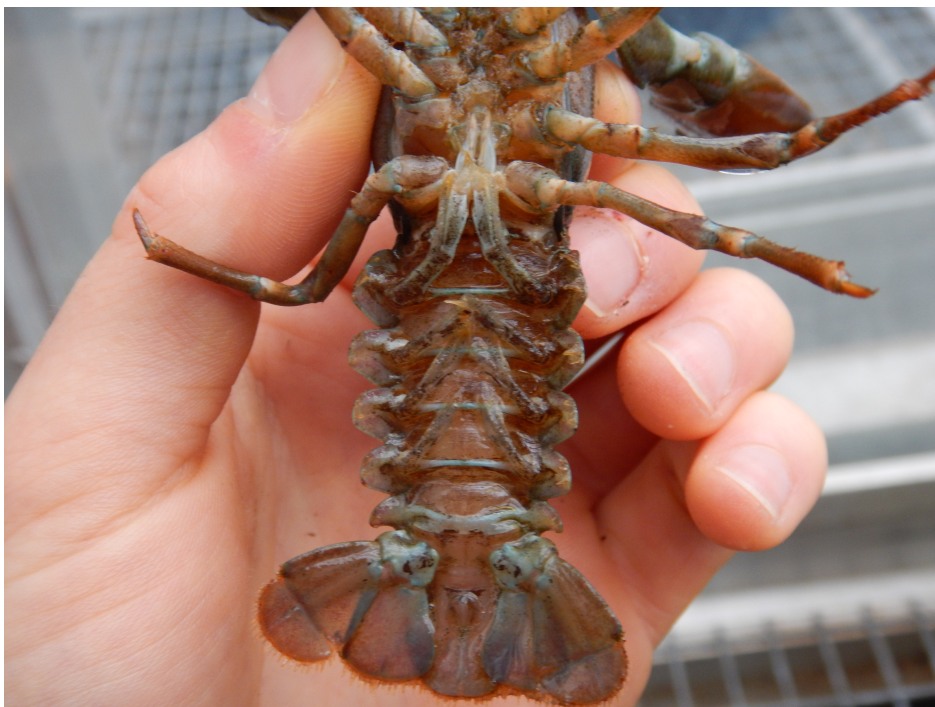

**(b)**

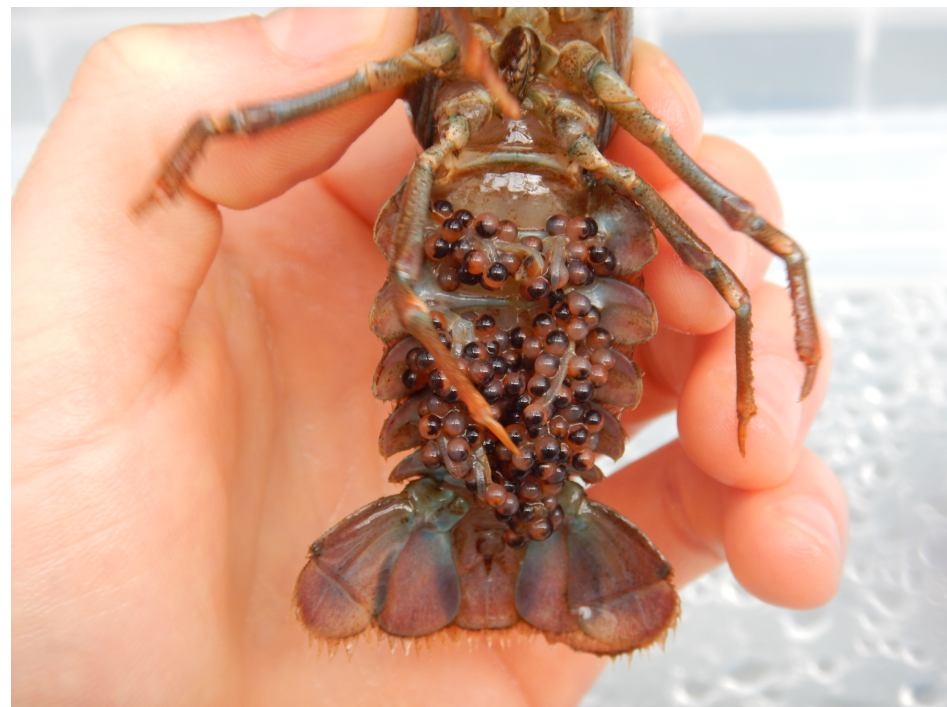

**(c)**

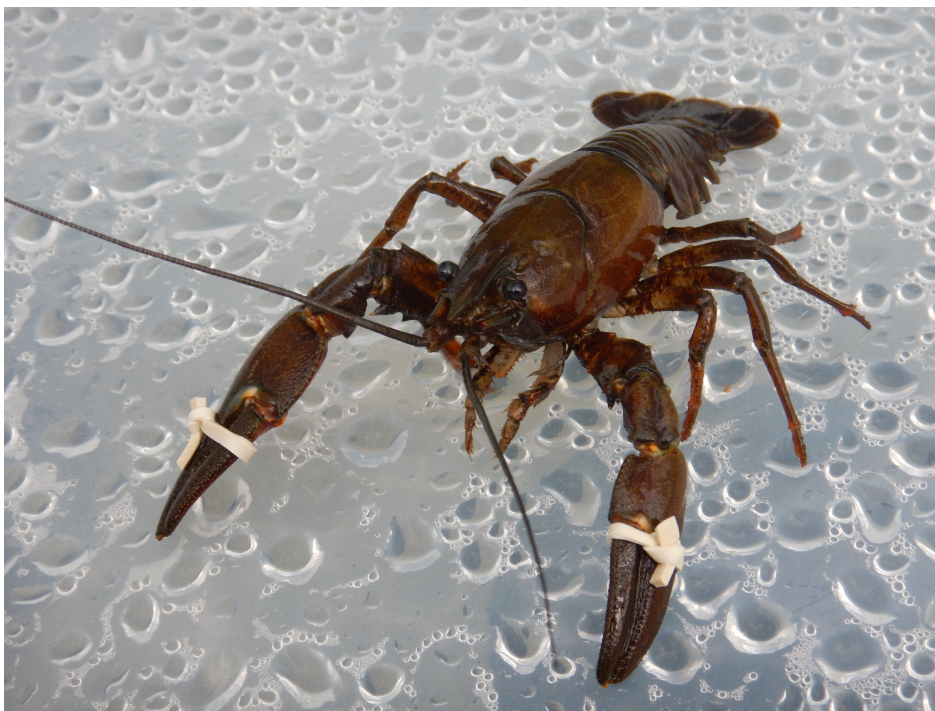

**(d)**

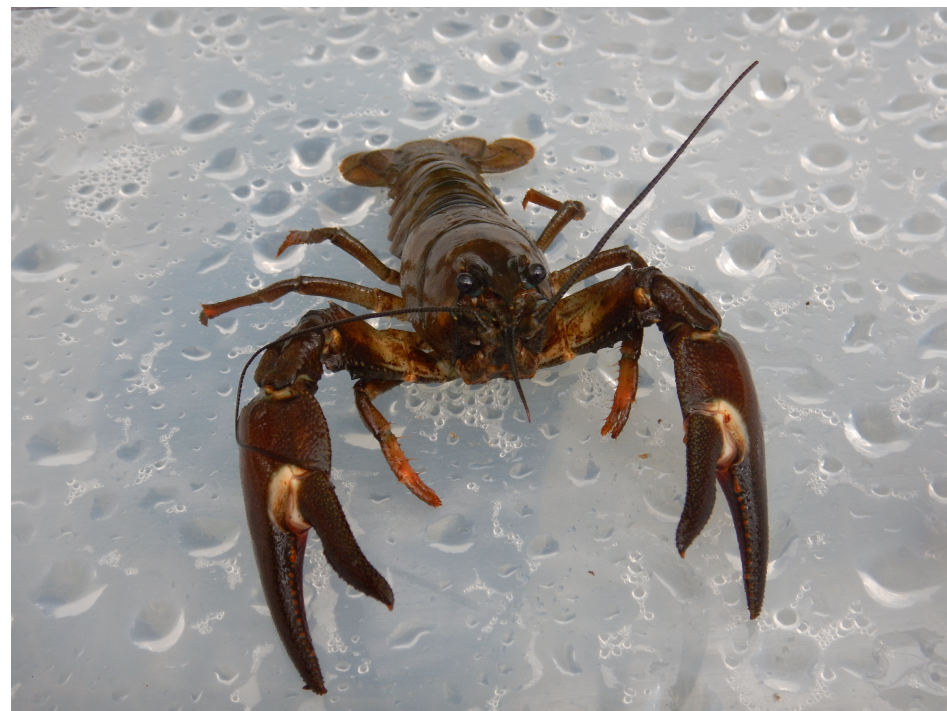

Supplement: Supplementary file 2 [file ECE3-7-7777-s002.pdf]

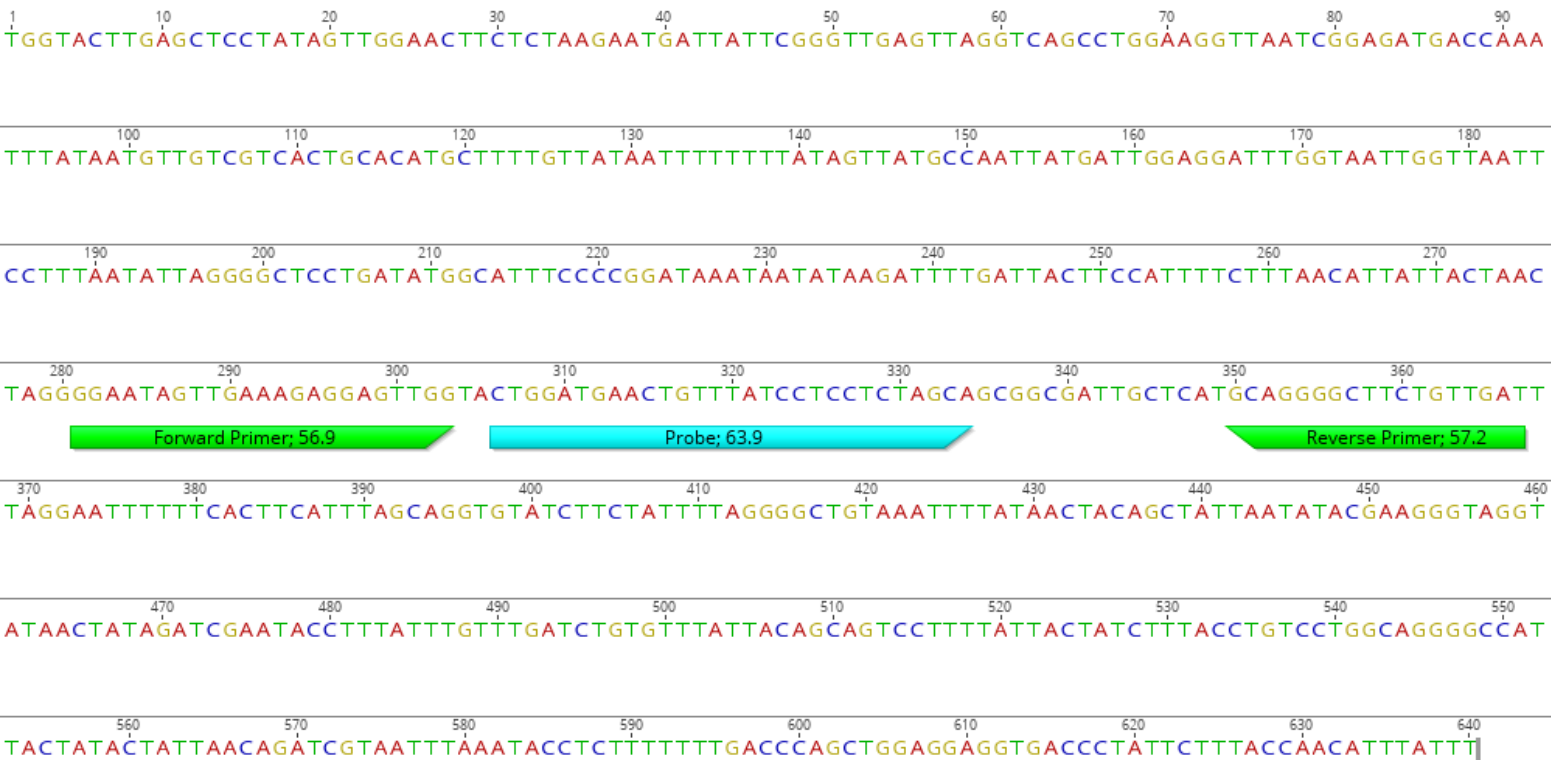

Supplement: Supplementary file 3 [file ECE3-7-7777-s003.pdf]

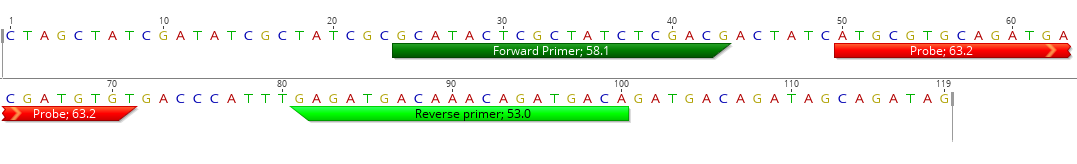

Supplement: Supplementary file 4 [file ECE3-7-7777-s004.PNG]

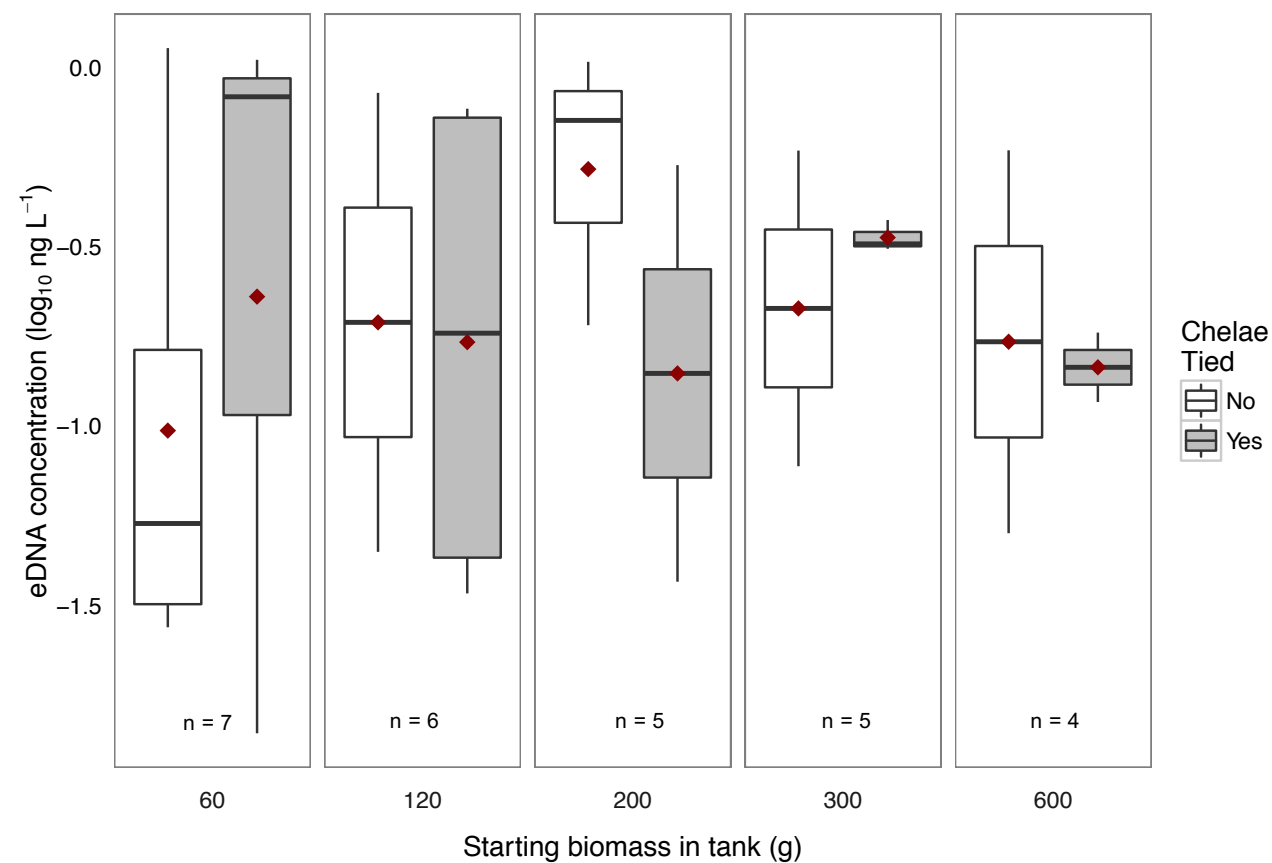

Supplement: Supplementary file 5 [file ECE3-7-7777-s005.pdf]

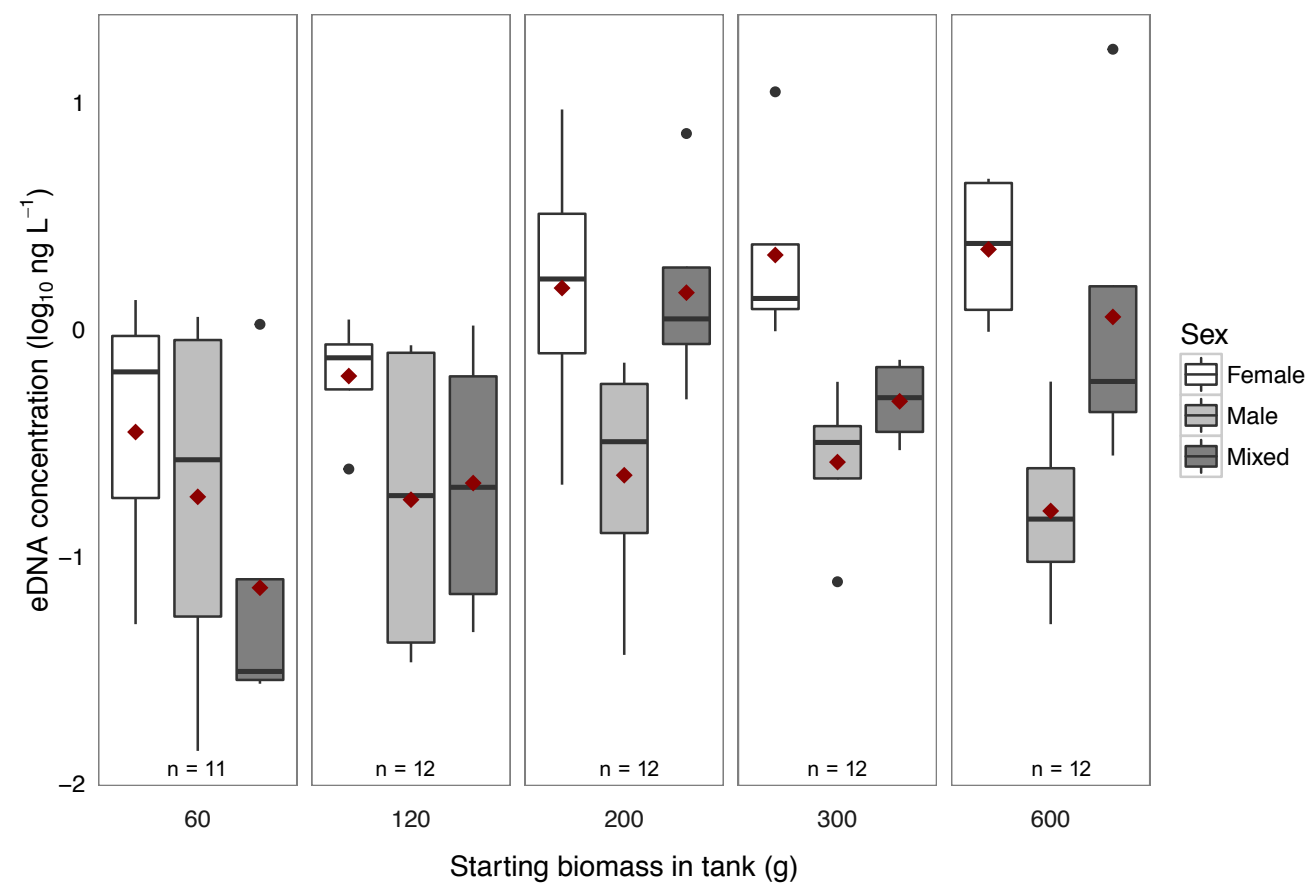

Supplement: Supplementary file 6 [file ECE3-7-7777-s006.pdf]
