# Supplementary material for: Behavior and season affect crayfish detection and density inference using environmental DNA
Source: Ecol Evol. 2017 Aug 24;7(19):7777–85. doi: 10.1002/ece3.3316 (PMC5632632; doi:10.1002/ece3.3316)
Supplement: Supplementary file 7 [file ECE3-7-7777-s007.docx]

Legend of supplementary figures

Fig S1. Maps showing the distribution of (a) *Austropotamobius pallipes* and (b) *Pacifastacus leniusculus* in Great Britain. Maps created in QGIS v2.16.3 (QGIS Development Team 2009) using data from the NBN Gateway https://data.nbn.org.uk/.

Fig. S2 Photos of *Pacifastacus leniusculus* showing (a) the underside of the tail of a male, (b) an ovigerous female with eggs attached to the pleopods, (c) an individual with tied chelae to prevent fighting and (d) an individual with untied chelae.

Fig. S3 Nucleotide sequence for *Pacifastacus leniusculus* Cytochrome *c* oxidase subunit 1 (COI) gene, NCBI Accession: KU603536.1 with primers and probe sequences with their melting points highlighted. Image created using Genious v10.0.7 (Kearse et al., 2012).

Fig. S4 Nucleotide sequence for the synthetic gene with primers and probe designed to test for qPCR inhibition testing. Image created using Genious v10.0.7 (Kearse et al., 2012).

Fig. S5 There was no significant difference in eDNA concentration between tanks in which crayfish had their chelae tied (grey boxes) and tanks in which they did not (white boxed) when tanks including ovigerous females were taken out of the dataset, red diamonds indicate mean eDNA concentration.

Fig. S6 Tanks containing only females (white boxes) contained significantly more eDNA than tanks with only males (light grey boxes) but not significantly more than mixed-sex tanks (dark grey boxes), red diamonds indicate mean eDNA concentration. There was no significant difference in eDNA concentration between male-only and mixed-sex tanks.
