# Supplementary material for: Behavior and season affect crayfish detection and density inference using environmental DNA
Source: Ecol Evol. 2017 Aug 24;7(19):7777–85. doi: 10.1002/ece3.3316 (PMC5632632; doi:10.1002/ece3.3316)
Supplement: Supplementary file 8 [file ECE3-7-7777-s008.docx]

Table S1. Sex-ratio, state of chelae and biomass for each tank with the concentration of amplified DNA in 1L of sampled water and the corresponding number of double-stranded copies.

| Tank | Run | Sex | Tied | Biomass (g) | Eggs present | Average Ct value | DNA in 1L sample (ng L^-1^) | DNA copies per L of sampled water |
| --- | --- | --- | --- | --- | --- | --- | --- | --- |
| 1 | 1 | Male | Yes | 61.30 | No | 36.2 | 0.186 | 1986 |
| 2 | 1 | Male | Yes | 122.30 | No | 36.3 | 0.179 | 1905 |
| 3 | 1 | Male | Yes | 200.60 | No | 36.7 | 0.131 | 1399 |
| 4 | 1 | Male | Yes | 304.10 | No | 37.4 | 0.080 | 854 |
| 5 | 1 | Male | Yes | 593.65 | No | 38.9 | 0.029 | 306 |
| 6 | 1 | Female | Yes | 60.60 | Yes | 35.5 | 0.298 | 3185 |
| 7 | 1 | Female | Yes | 128.90 | No | 36.4 | 0.162 | 1729 |
| 8 | 1 | Female | Yes | 195.60 | Yes | 35.7 | 0.274 | 2924 |
| 9 | 1 | Female | Yes | 279.65 | Yes | 35.5 | 0.305 | 3259 |
| 10 | 1 | Female | Yes | 586.47 | Yes | 33.9 | 0.892 | 9517 |
| 11 | 1 | Mixed | Yes | 64.90 | No | 35.9 | 0.238 | 2535 |
| 12 | 1 | Mixed | Yes | 93.82 | Yes | 36.8 | 0.126 | 1345 |
| 13 | 1 | Mixed | Yes | 184.87 | Yes | 33.4 | 1.329 | 14180 |
| 14 | 1 | Mixed | Yes | 310.00 | No | 37.2 | 0.092 | 983 |
| 15 | 1 | Mixed | Yes | 563.75 | Yes | 36.8 | 0.120 | 1281 |
| 16 | 1 | Male | No | 63.50 | No | 35.7 | 0.259 | 2761 |
| 17 | 1 | Male | No | 117.90 | No | 36.3 | 0.172 | 1840 |
| 18 | 1 | Male | No | 208.80 | No | 36.5 | 0.155 | 1651 |
| 19 | 1 | Male | No | 282.30 | No | 39.3 | 0.022 | 236 |
| 20 | 1 | Male | No | 488.08 | No | 36.8 | 0.125 | 1336 |
| 21 | 1 | Female | No | 64.80 | Yes | 36.5 | 0.157 | 1670 |
| 22 | 1 | Female | No | 125.30 | Yes | 37.7 | 0.075 | 800 |
| 23 | 1 | Female | No | 204.60 | Yes | 33.4 | 1.315 | 14037 |
| 24 | 1 | Female | No | 266.43 | Yes | 35.0 | 0.449 | 4795 |
| 25 | 1 | Female | No | 590.80 | Yes | 35.0 | 0.444 | 4734 |
| 26 | 1 | Mixed | No | 43.97 | No | 41.6 | <LOQ | - |
| 27 | 1 | Mixed | No | 124.20 | Yes | 37.4 | 0.078 | 828 |
| 28 | 1 | Mixed | No | 203.20 | Yes | 36.3 | 0.183 | 1957 |
| 29 | 1 | Mixed | No | 261.17 | Yes | 35.7 | 0.272 | 2898 |
| 30 | 1 | Mixed | No | 527.41 | Yes | 37.3 | 0.088 | 939 |
| 1 | 2 | Male | Yes | 63.20 | No | 38.6 | 0.014 | 148 |
| 2 | 2 | Male | Yes | 126.60 | No | 37.4 | 0.034 | 364 |
| 3 | 2 | Male | Yes | 203.20 | No | 37.3 | 0.037 | 392 |
| 4 | 2 | Male | Yes | 296.50 | No | 34.3 | 0.322 | 3438 |
| 5 | 2 | Male | Yes | 583.03 | No | 35.1 | 0.182 | 1945 |
| 6 | 2 | Female | Yes | 61.60 | Yes | 39.9 | <LOQ | - |
| 7 | 2 | Female | Yes | 118.90 | Yes | 32.7 | 1.103 | 11774 |
| 8 | 2 | Female | Yes | 202.70 | Yes | 34.9 | 0.207 | 2205 |
| 9 | 2 | Female | Yes | 294.79 | Yes | 32.4 | 1.322 | 14110 |
| 10 | 2 | Female | Yes | 587.10 | Yes | 32.4 | 1.311 | 13991 |
| 11 | 2 | Mixed | Yes | 60.60 | Yes | 37.7 | 0.029 | 308 |
| 12 | 2 | Mixed | Yes | 125.20 | No | 37.0 | 0.046 | 494 |
| 13 | 2 | Mixed | Yes | 210.00 | Yes | 32.6 | 1.189 | 12687 |
| 14 | 2 | Mixed | Yes | 278.63 | Yes | 34.7 | 0.293 | 3130 |
| 15 | 2 | Mixed | Yes | 609.20 | Yes | 33.3 | 0.693 | 7399 |
| 16 | 2 | Male | No | 63.60 | No | 36.2 | 0.085 | 912 |
| 17 | 2 | Male | No | 118.60 | No | 37.0 | 0.045 | 475 |
| 18 | 2 | Male | No | 200.50 | No | 35.1 | 0.191 | 2039 |
| 19 | 2 | Male | No | 300.90 | No | 34.7 | 0.250 | 2668 |
| 20 | 2 | Male | No | 598.20 | No | 33.5 | 0.587 | 6260 |
| 21 | 2 | Female | No | 57.20 | Yes | 36.8 | 0.050 | 536 |
| 22 | 2 | Female | No | 120.40 | Yes | 33.1 | 0.788 | 8407 |
| 23 | 2 | Female | No | 195.00 | Yes | 29.8 | 9.289 | 99124 |
| 24 | 2 | Female | No | 298.20 | Yes | 29.6 | 11.111 | 118569 |
| 25 | 2 | Female | No | 587.05 | Yes | 30.9 | 4.344 | 46360 |
| 26 | 2 | Mixed | No | 60.20 | No | 37.6 | 0.033 | 357 |
| 27 | 2 | Mixed | No | 121.00 | Yes | 32.8 | 1.038 | 11074 |
| 28 | 2 | Mixed | No | 203.80 | No | 33.1 | 0.817 | 8715 |
| 29 | 2 | Mixed | No | 300.30 | Yes | 33.2 | 0.733 | 7826 |
| 30 | 2 | Mixed | No | 597.20 | Yes | 29.1 | 17.111 | 182596 |
